# Motor-Evoked Potentials in the Lower Back Are Modulated by Visual Perception of Lifted Weight

**DOI:** 10.1371/journal.pone.0157811

**Published:** 2016-06-23

**Authors:** Frank Behrendt, Marc H. E. de Lussanet, Karen Zentgraf, Volker R. Zschorlich

**Affiliations:** 1 University Children’s Hospital Basle, Basle, Switzerland; 2 Research Department, Reha Rheinfelden, Rheinfelden, Switzerland; 3 Institute of Sport and Exercise Sciences, University of Münster, Münster, Germany; 4 Institute of Sport Science, Department of Kinesiology, University of Rostock, Rostock, Germany; University of Ottawa, CANADA

## Abstract

Facilitation of the primary motor cortex (M1) during the mere observation of an action is highly congruent with the observed action itself. This congruency comprises several features of the executed action such as somatotopy and temporal coding. Studies using reach-grasp-lift paradigms showed that the muscle-specific facilitation of the observer’s motor system reflects the degree of grip force exerted in an observed hand action. The weight judgment of a lifted object during action observation is an easy task which is the case for hand actions as well as for lifting boxes from the ground. Here we investigated whether the cortical representation in M1 for lumbar back muscles is modulated due to the observation of a whole-body lifting movement as it was shown for hand action. We used transcranial magnetic stimulation (TMS) to measure the corticospinal excitability of the *m. erector spinae* (ES) while subjects visually observed the recorded sequences of a person lifting boxes of different weights from the floor. Consistent with the results regarding hand action the present study reveals a differential modulation of corticospinal excitability despite the relatively small M1 representation of the back also for lifting actions that mainly involve the lower back musculature.

## Introduction

In social interactions, humans demonstrate a remarkable ability to effortlessly understand and interpret the behavior and intention of others. With regard to this topic, it has been found that within this process the visual perception of human movements activates a complex cortical network that involves visual processing regions [1–3] as well as motor intention [4, 5] or motor and somatosensory representations in the brain [6–13] which belong to the so-called mirror neuron system [14]. Direct electrophysiological recordings in monkeys [15–18] and humans [19] have shown a discharge in such mirror neurons during execution and perception of actions.

The mere observation of actions is known to modulate motor excitability, which can be assessed by TMS [20–22]. TMS is a valuable, noninvasive tool to stimulate small brain regions and thereby to measure motor activation during the observation of other’s action. It was shown that parts of M1 that control particular muscles become increasingly facilitated during the mere observation of actions [20, 21]. It can be used to monitor changes in corticospinal excitability that specifically accompany motor performance [23] by measuring the amplitude of motor evoked potentials (MEPs) in the electromyogram recorded from the target muscle.

Such changes in the corticospinal excitability are specific for the muscles involved in the observed movement [24–28] and have revealed a shared temporal representation between the execution and observation of the same action [29–31]. In one paradigm the subjects observed reach-grasp-lift actions with MEPs elicited in three forearm/hand muscles [32] or abduction/adduction movements of the right index finger [33]. These studies found that the modulation of the MEPs directly corresponded with the grip force needed for holding and lifting the objects of various weights or differed in terms of force requirements of the finger abduction/adduction movement, respectively. Alaerts et al. [32, 34] and Helm et al. [33] thus showed that even the muscular force requirements of observed actions are reflected in the TMS-induced MEPs and concluded that the force requirements are encoded also in M1. There are indications that further features of actions are represented on a cortical neural level, such as accuracy affordances [35, 36] and effort [37].

People can accurately judge the weight of a box lifted from the ground by another person [38–41]. It has been suggested that the perception of the force requirements needed to visually judge the lifted weight are derived mainly from kinematic cues [42, 43]. This process also requires an inference of the force to be produced by the back musculature, i.e., the ES, which is strongly involved in such a whole-body lifting movements [44]. de Lussanet et al. [40, 41] have shown that such a sensorimotor, but not visual, judgment can be impaired in patients suffering from chronic low back pain or chronic shoulder pain which indicates that there is a link between chronic pain and the recognition of other people’s actions.

It is well-established that the ES and other lower back muscles have smaller sensorimotor representations compared to the hand musculature [45] which primarily refers to the size of the corresponding cortical surface. This is, inter alia, also reflected by the large difference in tactile acuity between the skin of the hand and the back. See Catley et al. [46] for a comparison using two-point discrimination tasks. Cortical representations of muscular movements are furthermore known to be much more detailed for muscles involved in precise and fine movements than for those involved in simpler movements. The large muscles of the back which merely maintain the upright posture are less physiologically endowed than the small muscles of the hands or the face. Additionally, the projection of the ES was found not to be as powerful for example, as that described in the intrinsic hand muscles [47]. Given on the one hand that the action observation process partly integrates necessary motor information and on the other hand that there is such a comparatively small representation of the back, it is unclear whether to expect a similar TMS-induced MEP modulation in the lower back musculature as compared to Alaerts et al. [32] due to the observation of a lifting movement.

But nonetheless, the ability to infer the goals or the intentions of observed actions is very robust which is necessary to successfully interact with others. It has been suggested that the process of recognising such goals can be understood within the so-called predictive coding framework [48] which states that the brain continually generates models of observed actions by minimizing the prediction error though recurrent or reciprocal interactions among levels of a cortical hierarchy that are engaged during action observation. It is considered an efficient way of processing the wealth of incoming information based on previous experience and also to infer motor intention.

However, as action understanding is a vital capability and as there is a direct link between motor control and the visual perception of human movements we hypothesized that the corticospinal excitability of the lower back musculature should be detectably modulated with the observation of visually presented lifting movements. Given that TMS is well-known to be an appropriate technique to assess corticospinal excitability modulations at the level of M1 [22, 49], it was used in the present study to explore excitability modulations during the observation of lifting movements that involve the lower back on a large scale. We expected that the muscular force requirements of the lower back are reflected in the MEPs measured in the ES.

## Materials and Methods

### Subjects

Fourteen healthy subjects (24.5 years; three females) gave written, informed consent prior to participation. Ethical approval was given by the local ethics committee of the medical faculty at the University of Rostock, Germany, in accordance with the Declaration of Helsinki.

### Visual stimuli

Point-light biological motion was used as visual stimulus, which does not contain image information but can nevertheless easily be recognized [50]. The perception of motion via point-light displays activates motor- and somatosensory representations in the brain [3, 8, 11, 51]. These brain regions are thought to hold a central role during motion perception as there is an activation during action execution and during action recognition [8, 14, 16, 52]. Depending on the task, the observation of point-light biological motion does not only activate these cortical networks but can also modulate spinal reflexes such as the gain of cutaneous reflex in *m. tibialis anterior*[53, 54].

In order to construct the point-light stimulus, 3-D recordings (Qualisys, Gothenburg, Sweden) of the main joints of a male actor lifting boxes were fed into an iBook computer running an in-house programmed application (MotionViewer 48, using XCode 3.1 and OpenGL). The boxes (32x27x32 cm) with side-grips for holding were of three different weights (3.25 kg, 12.5 kg and 22.25 kg). The actor was not aware of the weight of the box prior to lifting. A screen capture video showing the three experimental conditions can be found within https://osf.io/xk7na/.

**Fig 1:**
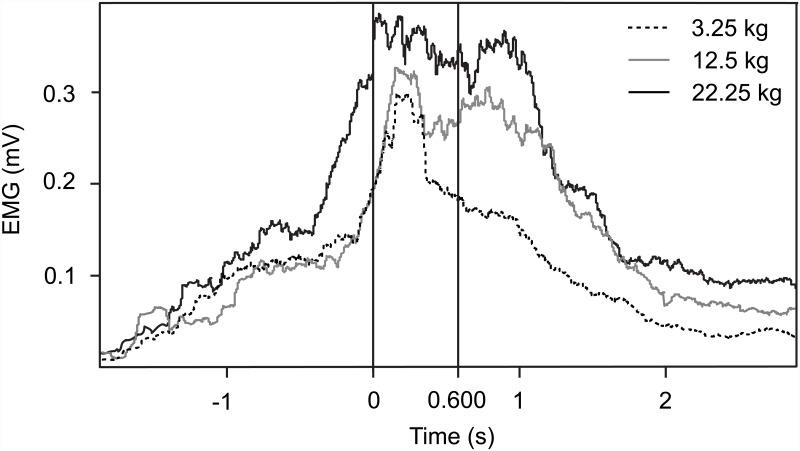
ES muscle activity in the actor who lifted the weights. Recorded by bipolar EMG over the lumbar back musculature (ES at 1st lumbar vertebra) for lifted boxes of 3.25, 12.50 and 22.25 kg. The vertical line at 0 s is the time when the box started to move (lifting onset). The second line (600 ms after lifting onset) represents the time point where the EMG activation was maximally separated as a function of weight, and therefore, this was the time that TMS was triggered.

The recorded movements were synchronized with respect to the onset of the movement of the box. Fig 1 shows the actor’s rectified EMG responses of the lifting movements of the three different boxes of the same recordings that were used as visual stimuli in the subsequent experiment. TMS stimulation was applied at 600 ms, when the movements were approximately half through and when the actor’s EMG responses were maximally separated between the different weights.

### Electromyographic recordings and TMS

The magnetically elicited MEPs were recorded with an EMG differential amplifier (biovision Wehrheim, Germany) and a gain factor of 500–1000 depending on the individual electrode-skin-interface condition. The amplifier input resistance was 10 G*Ω* and the bandwidth of 1 Hz to 1000 Hz. EMG-signals were further recorded with a DAQ-Card 6024 (National Instruments, Austin, Texas, USA) with a 12-bit resolution and 10000 samples per second, using DIAdem 8.1 (National Instruments, Austin, Texas, USA) signal acquisition program. A three second measurement period has been triggered in response to the demonstrated point-light-figure with a 1 second pre-trigger interval to the TMS and a post-trigger phase of 2 seconds to control full relaxation of the recorded muscle.

The EMG raw signals from the ES at 1st lumbar vertebra were high-pass filtered with a 2nd order digital Butterworth filter [55] with a cutoff frequency of 5 Hz before further calculation. The reflex responses were registered with three Ag-AgCl cup electrodes (Hellige baby-electrodes; GE medical systems, Milwaukee, USA) with an electrode surface area of 3 mm^2^ which were placed with a distance of 1 cm longitudinally over the belly of the muscles. The skin preparation was as follows: the skin was cleaned with alcohol and the hair was removed prior to electrode application. The skin abrasion generally results in a skin impedance lower than Z = 10 k*Ω* at 30 Hz. Contact between skin and electrode was provided with an electrode gel (Parker Laboratories, Fairfield, USA). Electrodes and twisted cables were fixed with self adhesive tape on the skin.

A magnetic stimulator R30 MagPro with MagOption (MagVenture, Skovlunde Denmark—formerly Medtronic) and a figure of eight coil type D-B80 were used for TMS. The stimulator generated symmetric pulses of 290 *μ*s duration. The coil was first placed 1cm lateral to the vertex over the right hemisphere on the antero-posterior level of the vertex in order to find the individual hotspot. After the process of searching for the most sensitive position the handheld coil was then fixed via a mechanical adjustable Manfrotto (Feltre, Italy) arm. The figure of eight coil (coil angle 45°) was oriented so that the induced electric current flows in a medio-lateral direction over the right motor cortex. The stimulation intensity (maximal stimulator output) was defined as 120% of resting motor threshold to reach detectable MEP on the ES which is in line with other studies that measured TMS-induced MEPs recorded from the relaxed lumbar ES muscle [56, 57]. The average stimulator output was 63.9% (SD 9.4%). Motor threshold in turn was defined as stimulus intensity that produced a MEP of more than 100 *μ*V in 3 of 5 trials. The magnetic gradient therefore lies in the range between 50–70 A/*μ*s. Regarding the advantage of a greater differentiation in a poorly represented muscle area TMS was carried out in biphasic mode [4, 58, 59].

### Procedure

With the head restrained in a chin-forehead rest the participants were seated in an upright position in front of a 23” TFT display connected to the iBook. Via the USB port, the iBook was also connected to the magnetic stimulator. By this, the presentation software triggered the electrical stimulation.

The visual stimulus presented a slightly oblique frontal view of a point-light figure that did one single step towards a box (depicted by 8 white dots) on the ground, lifted the box towards his chest and placed the box down on the floor in front. The three conditions of the experiment comprised the lifting of three different boxes, whereas the boxes only differed in weight but not in size. Each of the three different sequences was presented 20 times in random order. During each sequence, a single TMS pulse was delivered 600 ms after the beginning of the lifting phase. This time was selected because it was in the middle of each ongoing movement, and the actor’s EMG differed maximally between the three weights (Fig 1). In order to get familiarized with the visual stimuli, the subjects were shown the sequences before the experiment started.

Additionally, after each lifting sequence all subjects were asked to verbally judge the lifted weight within a range of 0-25 kg. This was planned to control whether the participants are able to discriminate the weights in that task. A bad performance in the weight discrimination task was not considered an exclusion criterion. All subjects were able to perceptually discriminate the different weights. In order to give a rating the point-light display disappeared and was replaced by a response display. This response display consisted of a slider, controlled by horizontal movements of the mouse, that pointed at a horizontal, continuous scale (about 18 cm long) marked with ticks and the numbers 05-10-15-20 and 25 kg. The slider was adjusted by the experimenter according to the verbal judgment of the subject. Once the slider had been moved, a press of the space bar button confirmed the response setting and started the display for the next trial after a 4-second pause. The procedure ensured a minimum interstimulus interval between two successive TMS-pulses of at least 10 seconds.

### Control Experiment

Background EMG during the visual perception of the lifting sequences was assessed in a control experiment without TMS. This was planned to determine a potentially confounding effect of condition-specific background EMG-modulations. For that purpose we additionally tested further 11 subjects (24.4 years, 7 females) using the same experimental setup without eliciting MEPs. Mean EMG activity of a one second time window beginning with the start of each observed lifting sequence was calculated.

### Data Analysis

Prestimulation EMG was assessed as it is well-known to directly influence MEP amplitudes [60]. For this, the root-mean-square values across a 50 ms time window before each stimulation were computed as well as the mean and standard deviation of the EMG background scores over all trials for each subject separately. Trials were removed when the background-EMG exceeded the mean + 2.5 standard deviations.

As in a few cases polymorphic MEPs were obtained, the integrals of the filtered surface EMG data of the MEPs were used for analysis. All polymorphic MEPs were included in the analysis. The interval of the integral calculation was set to 15-45 ms after the beginning of the stimulation as all MEP responses lay in this time window. The MEP integral values were then normalized by computing the standard scores (Z-scores) using the individual mean and standard deviation of the integral MEP data for the normalization of the data of each subject.

## Results

### MEP data

**Fig 2:**
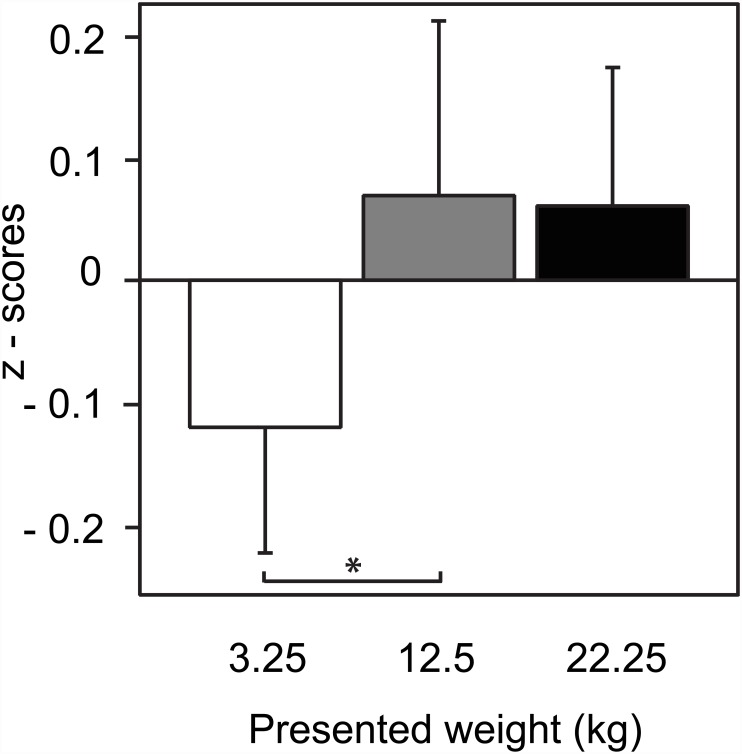
Z-scores of the averaged MEP integral values. Recorded in all subjects from ES in response to visually presented lifting of boxes of 3 different weights from the ground. Error bars represent the within-subject 95% confidence intervals.

The average normalized data over all subjects are shown in Fig 2. The repeated measures ANOVA on the Z-scores of the MEP integral data revealed that the main effect *weight* as within-subject factor was statistically significant (F_2,26_ = 4.141, *p* = 0.016, $\eta_{p}^{2}=0.015$). *Fisher’s PLSD post-hoc* analysis on the Z-scores showed that MEP responses were significantly smaller for observing the lifting of the lightest box as compared with observing the heavier boxes being lifted with *p* = 0.013 and *p* = 0.017 for 12.5 and 22.25 kg, respectively.

### Control experiment

The EMG data of the control experiment without TMS were also subjected to a repeated measures ANOVA. A differential muscular activity in the ES could not be found throughout the conditions as the main effect *weight* was not statistically significant (F_2,20_ = 1.298, *p* = 0.295, $\eta_{p}^{2}=0.115$). This indicated that there were no condition-specific modulations and thus no confounding modulations in the background EMG that might have had an impact on the MEPs.

### Weight judgment

**Fig 3:**
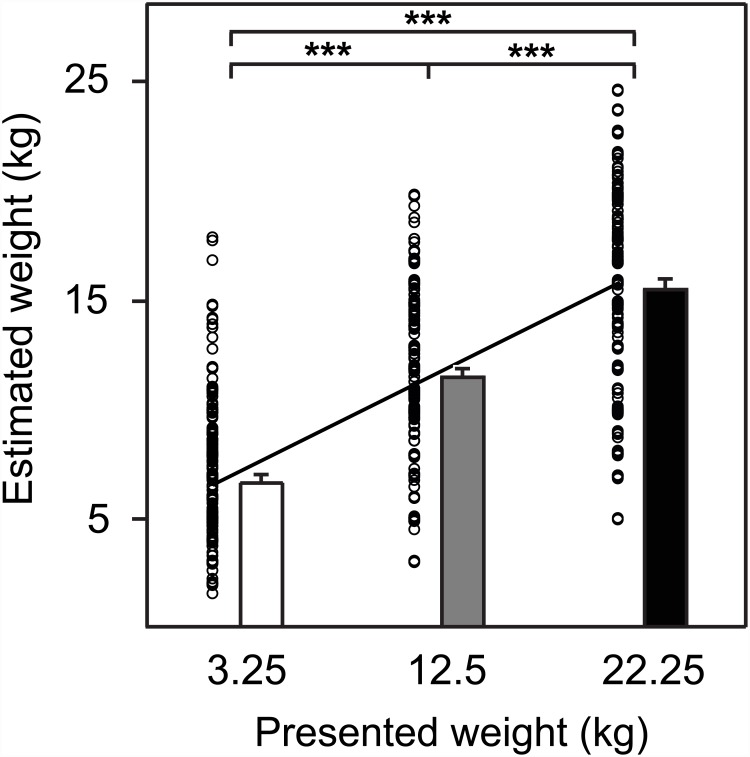
Average estimated weight of all subjects over the 3 conditions. The single judgments are displayed, as well as the group regression line. The weight discrimination, computed as regression slope, was 0.47.

The subjects ability to discriminate between the weights in the presented point-light displays was also tested (Fig 3). The data of a further repeated measures ANOVA on the judged weights showed a significant main effect (F_2,26_ = 536.878, *p* < 0.0001, $\eta_{p}^{2}=0.664$). Significant differences were also found between all 3 conditions using *Fisher’s PLSD post-hoc* tests (*p* < 0.0001), which confirmed that the subjects were well able to recognize the differences in the force requirement and hence the lifted weight.

## Discussion

Research on the observation of actions has repeatedly provided evidence for a significant modification of the motor system when a subject observes an action performed by another individual [21, 24], even when the action is depicted by a point-light figure [3, 8, 11, 51]. Humans are also well able to recognize movement specific effort when it can only be derived from the displayed kinematics of such a point-light figure, for example, when lifting objects [38, 39, 50]. This ability is likely partially based upon the same neural processes that lead to motor excitability modulations during action observation which can reflect several features of observed movements, including the force requirement [32]. Their findings indicated not only that modulations in the corticospinal excitability during action observation can be especially related to the muscles involved in the observed action, but that these changes also specifically reflect the muscular force requirements. However, in the study by Alaerts et al. [32] corticospinal excitability data were obtained by stimulating in the comparatively extensive cortical hand area of M1.

In this context, the present experiment tested whether the observer’s motor system reflects the force requirements of observed actions that mainly involve the lower back with its small cortical representations. Indeed, we found a modulation of MEPs in the ES due to observing such actions. Insofar, the present results extend the findings by Alaerts et al. [32]. As we have applied TMS to such a small representation in primary motor cortex and due to the accordingly less targeted cortical control of the back musculature, both might have influenced the observed effect in the present study.

The elicited corticospinal responses only slightly differed between the 12.5 kg and 22.25 kg observation conditions with no statistically significant difference. Such a saturation or ceiling effect has been observed before [32]. Thus it might be cautiously hypothesized that this ceiling effect is reflected by M1 facilitation when movements are observed which imply relatively high force requirements, although weight discrimination did not exhibit a similar effect. Further possible explanations could be that the effect might be related to non-linear properties of motor unit recruitment or it might simply reflect random variation in the signal.

The setting of the TMS-experiment obviously did not hamper a proper weight judgment by the subjects. On average they did not perfectly judge the weight of the manipulated objects, but their performance was comparable to the performance of subjects in other studies with point-light stimuli [38, 39, 61] and with filmed stimuli [39, 62, 63]. Therefore, no data of any of the subjects had to be excluded from further analysis.

The control experiment did not reveal any influence of the mere observation of the stimuli on the background EMG without applying TMS. As expected we could not find a condition-specific modulation in the ES-EMG during the observation of box lifting. In earlier studies using point-light biological motion we could not find a systematic modulation either [53, 54]. There are further studies that could not detect a potential effect of action observation on EMG as well which is in line with our results [64, 65]. However, the latter studies used different movements and stimuli as compared to the current experiment. For this reason it was necessary to include the control experiment to clarify this issue for the used paradigm.

Although speculative, the findings and the underlying processes could be embedded in the predictive coding concept [66] which has been validated by a number of brain imaging studies [67–71]. In order to comprehend the goal or the specific physical constraints of an action, such as the weight of a box being lifted, the observer can just rely upon her or his own sensory experiences related to the kinematics of the observed action. The automatically generated cortical activation pattern was proposed to reflect this process of mentally simulating the observed action [72]. The fact that observing biological motion triggers a modulation of motor-evoked potentials in the muscles involved in the corresponding effector [20], as also found in this study, was interpreted as reflecting incipient ideomotor capture [73] which means that the mental concept of an action unconsciously produces an automatic facilitation of motor circuits. The arousal of such an according concept fits well into the predictive coding as it is probably part of the process of inferring the intentions that drive observed actions. This continuous process of comparing predicted and observed kinematics generates a prediction error which is again fed back into the prediction process and which enables the observer to make an increasingly precise inference of the goal, of the agent’s motor commands and thereby likewise of the force requirements. Obviously, the time given from the start of the lifting sequence until the triggering of the TMS-pulse in our study was sufficient to address the according mental concept and to run the prediction process to unconsciously distinguish between the different motor commands related to the different boxes of different weights.

It is generally assumed that the lower back musculature mainly functions in terms of postural control and is in some degree not under direct cortical influence but is further driven by lower, subcortical control units. In this context it is quite surprising that such clearly adapted MEPs could be found in this study. The branched cortico-spinal pathway structure underlying the measured MEPs likely includes these lower control units of the lower back musculature. Hence, an influence of subcortical structures on the characteristics of the measured MEPs can not be excluded.

Our results provide evidence that MEP thresholds of even weakly represented muscle groups such as the lower back muscles can be modified by passive visual observation of actions. From the perspective of action understanding such a representation makes sense for the judgment of lifted weight because of the central role that these muscles play. It is also consistent with our earlier findings that action observation modulates reflex gains in peripheral body parts [53, 54]. However, the experiment does not include a baseline condition and it is therefore not possible to conclude a mirror-like facilitation effect from the data.

As chronic back pain patients are impaired in the sensorimotor judgement of such observed actions [40, 41] it would be of interest to also investigate the cortico-spinal excitability in these patients using a comparable paradigm to further elucidate the character of motor control impairment in chronic back pain patients.
